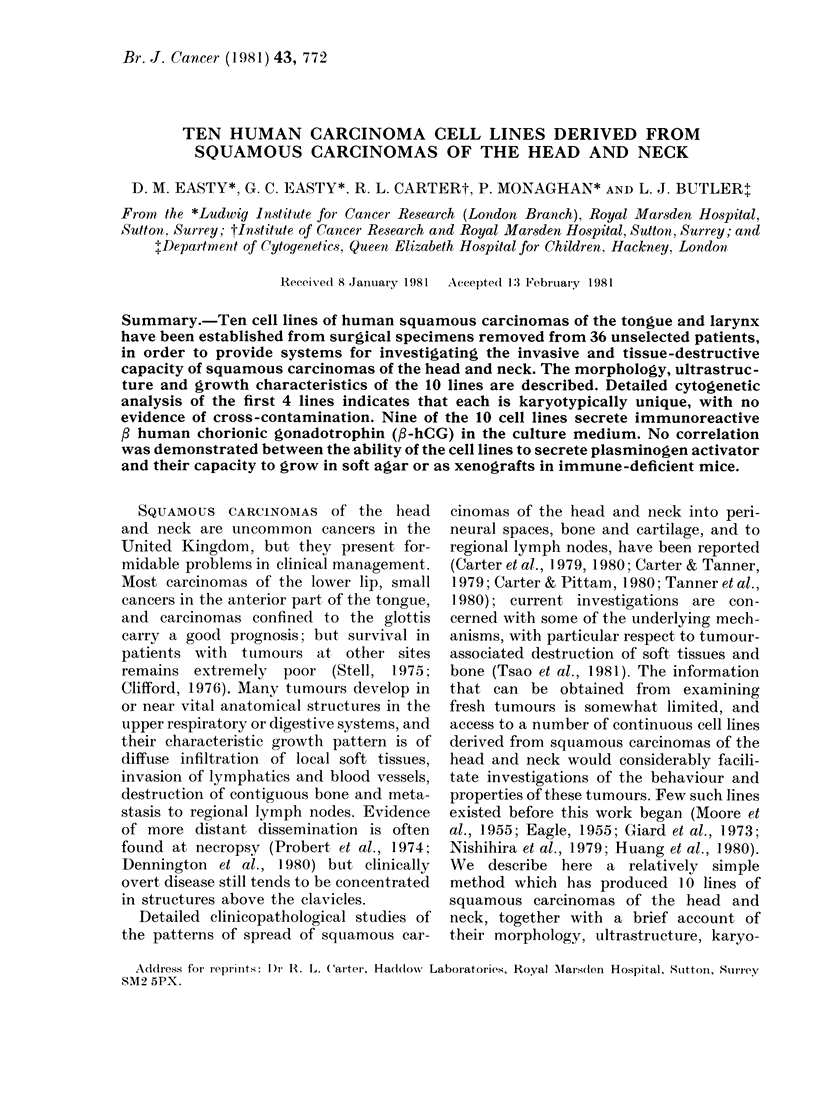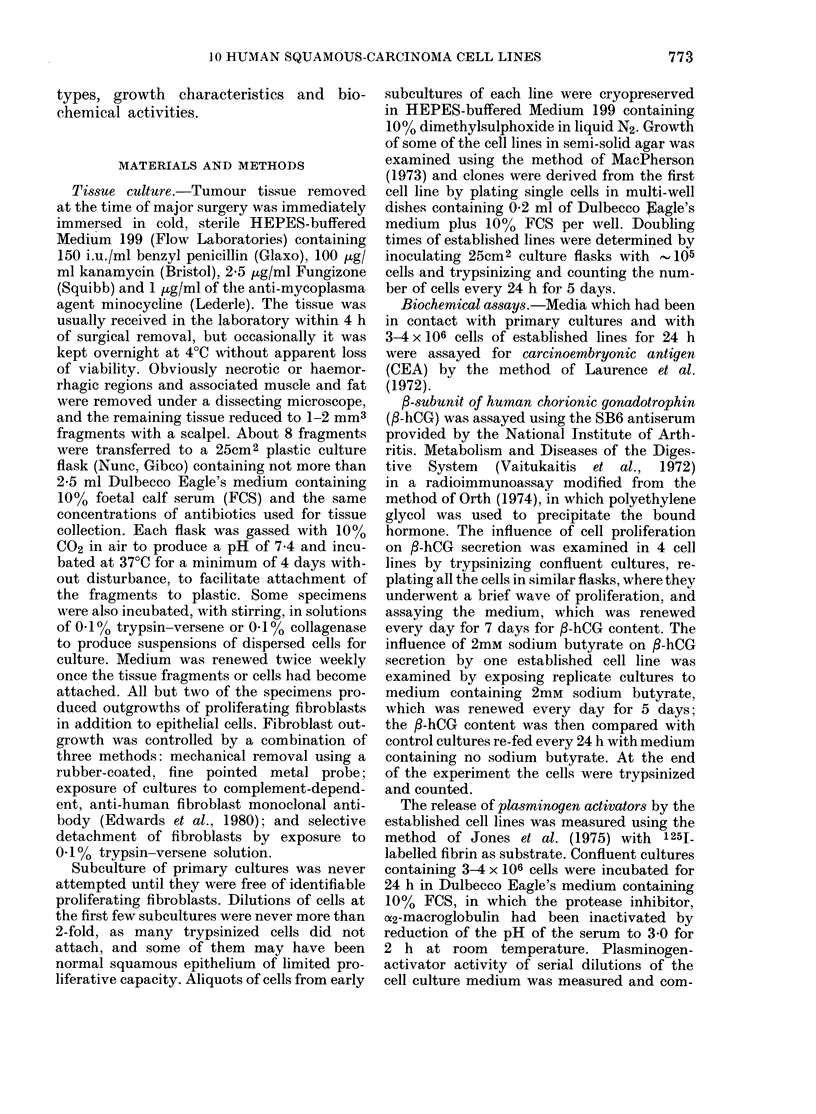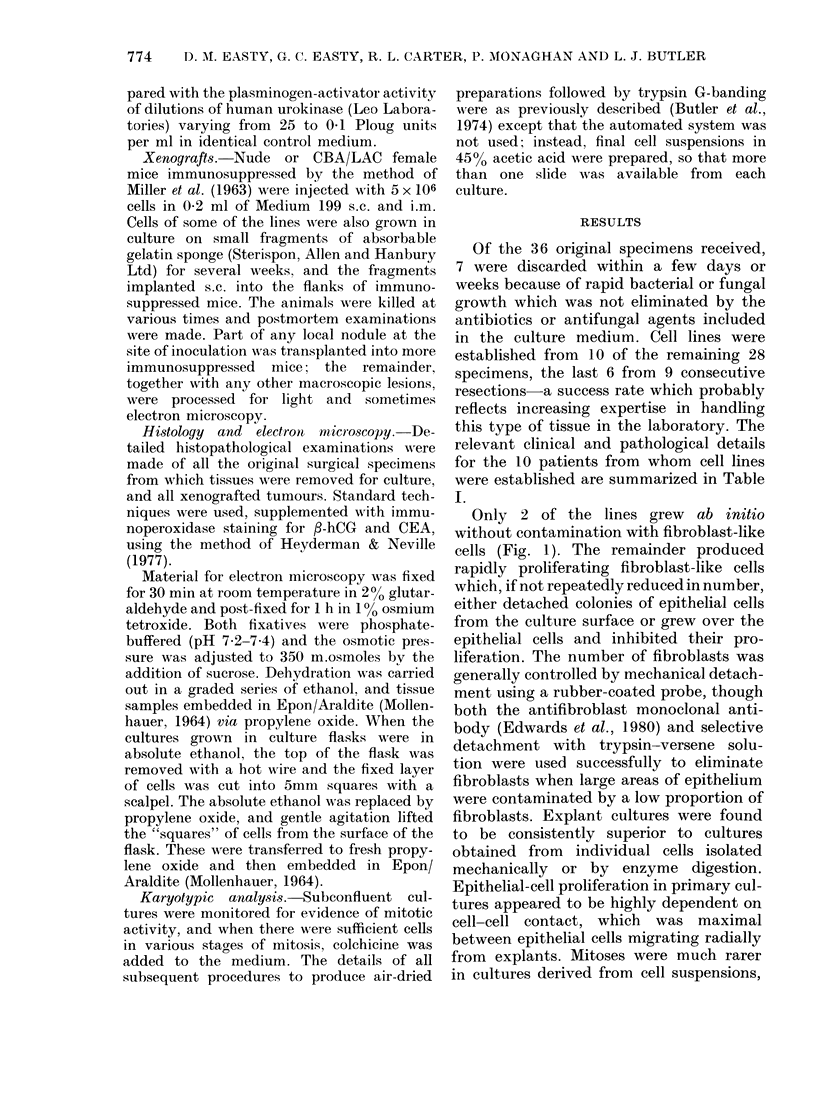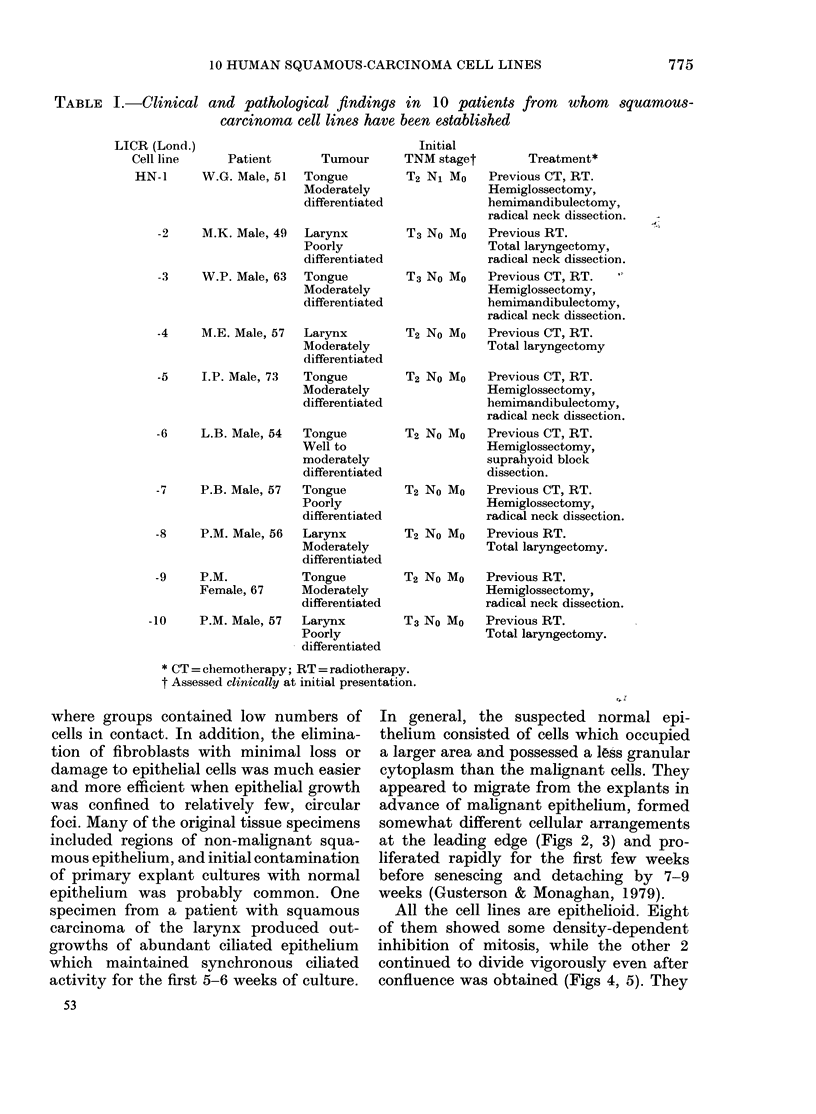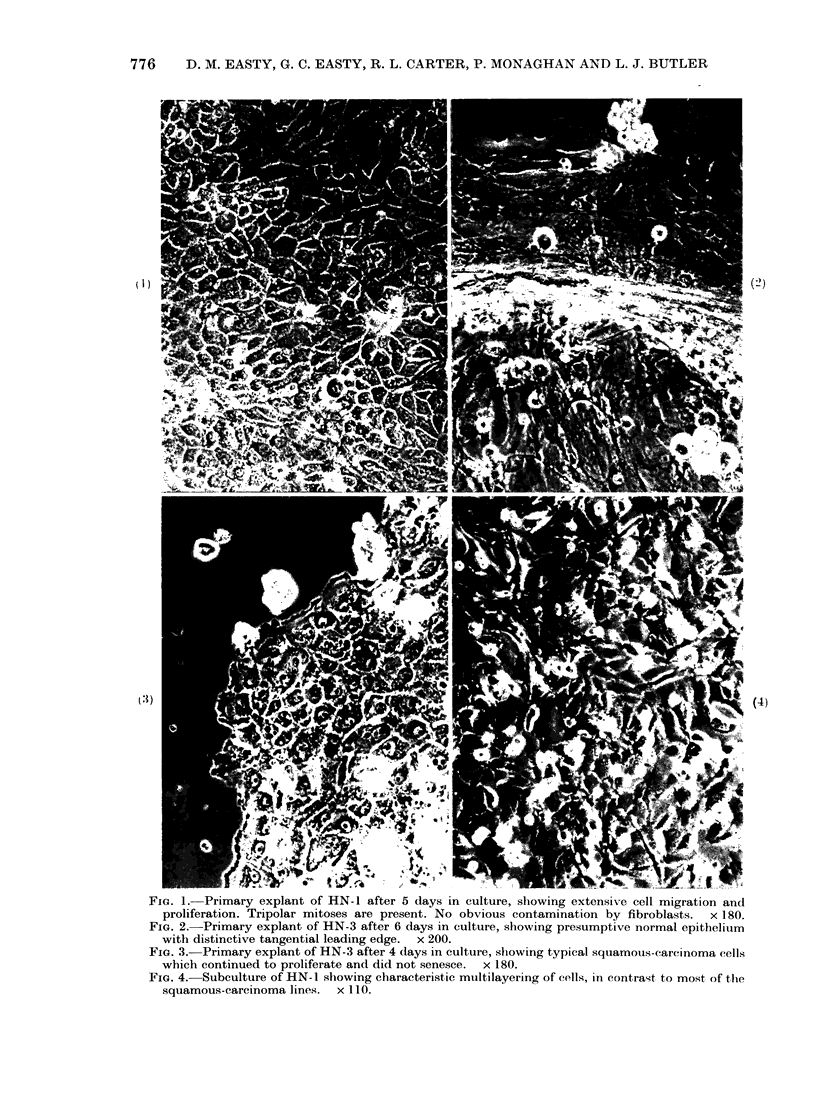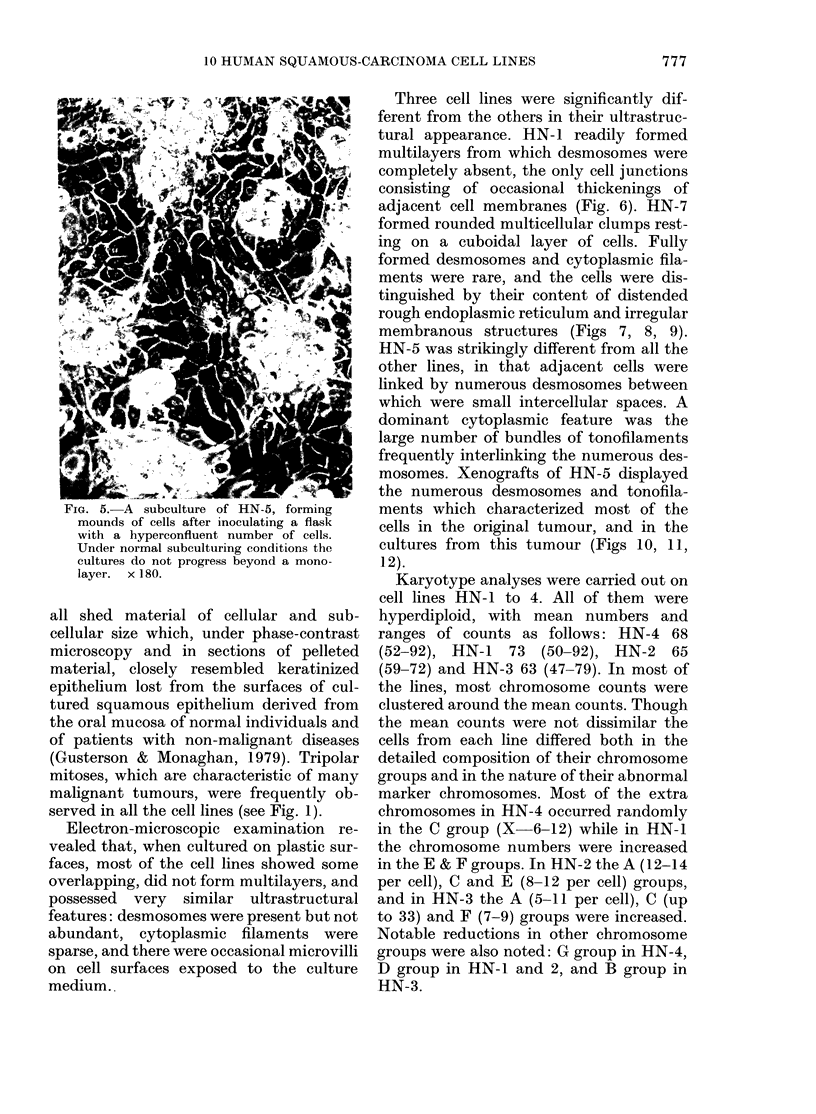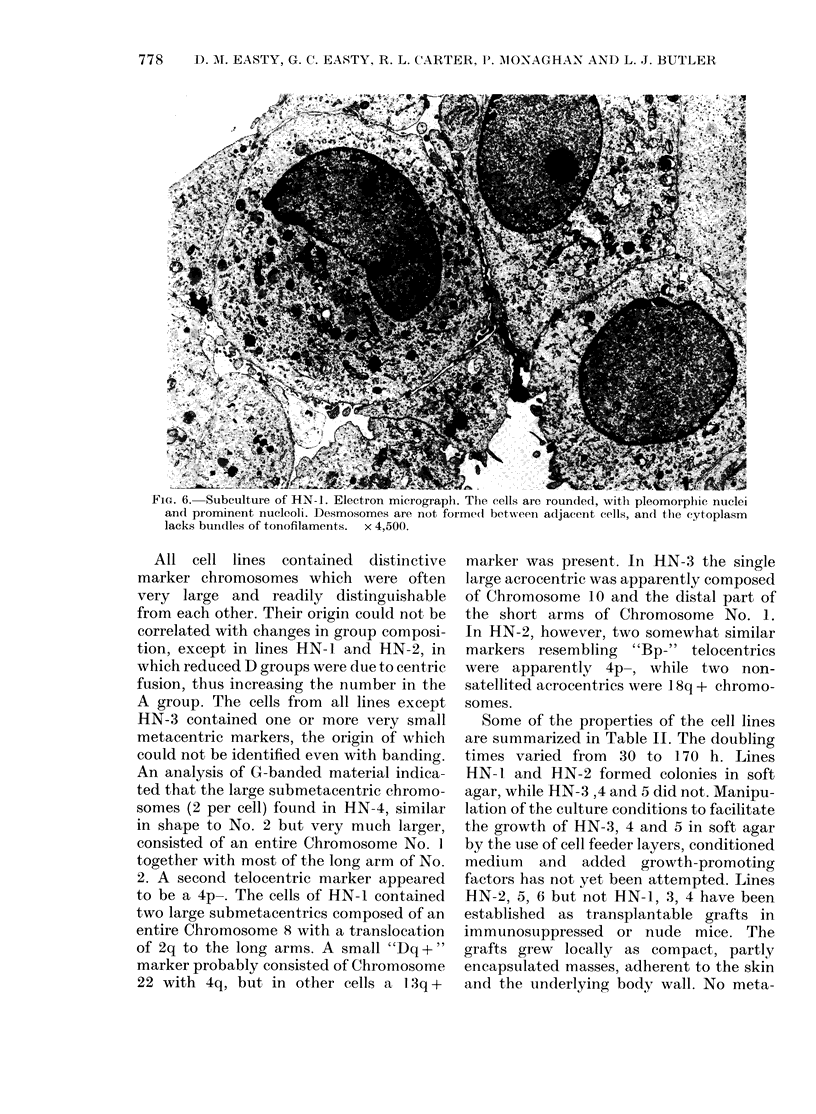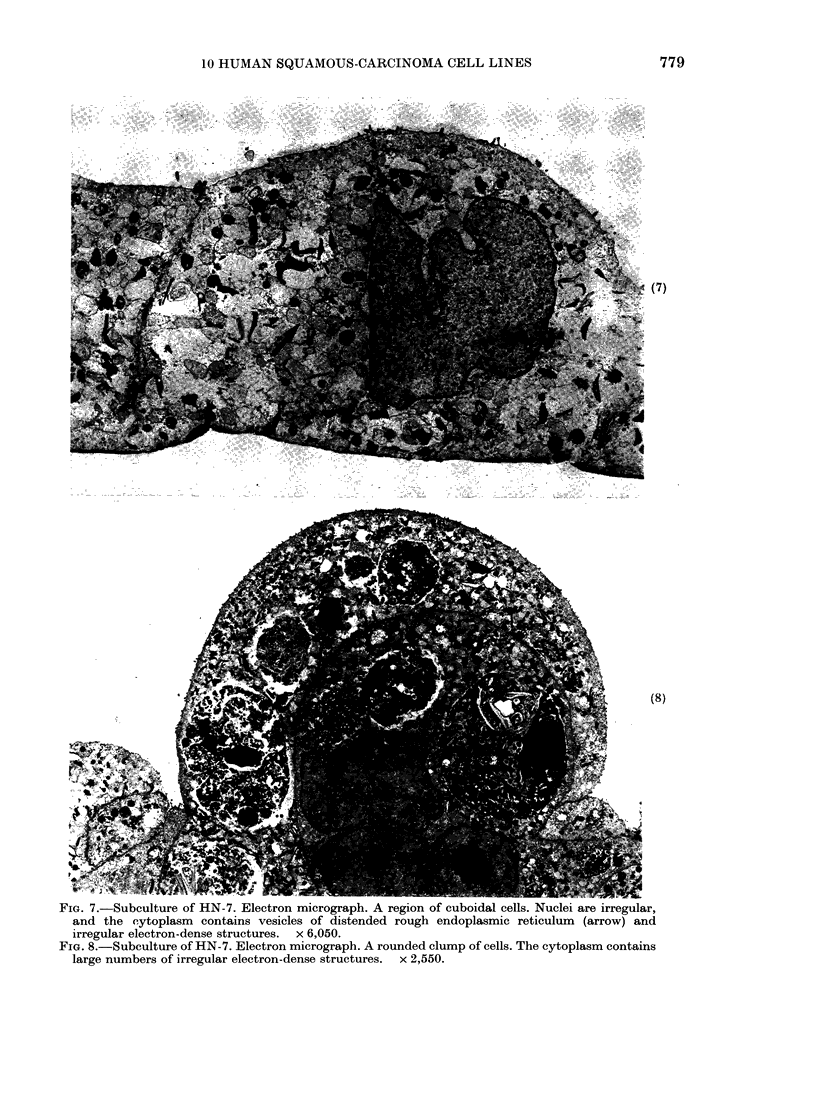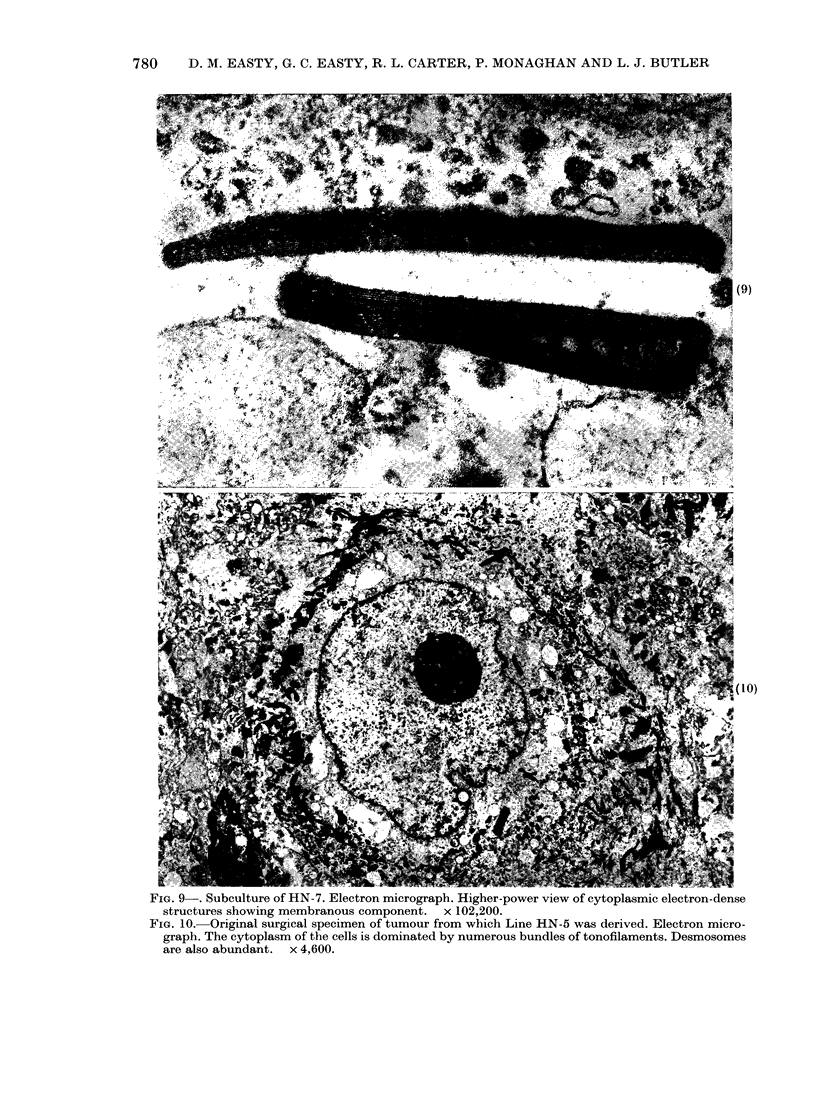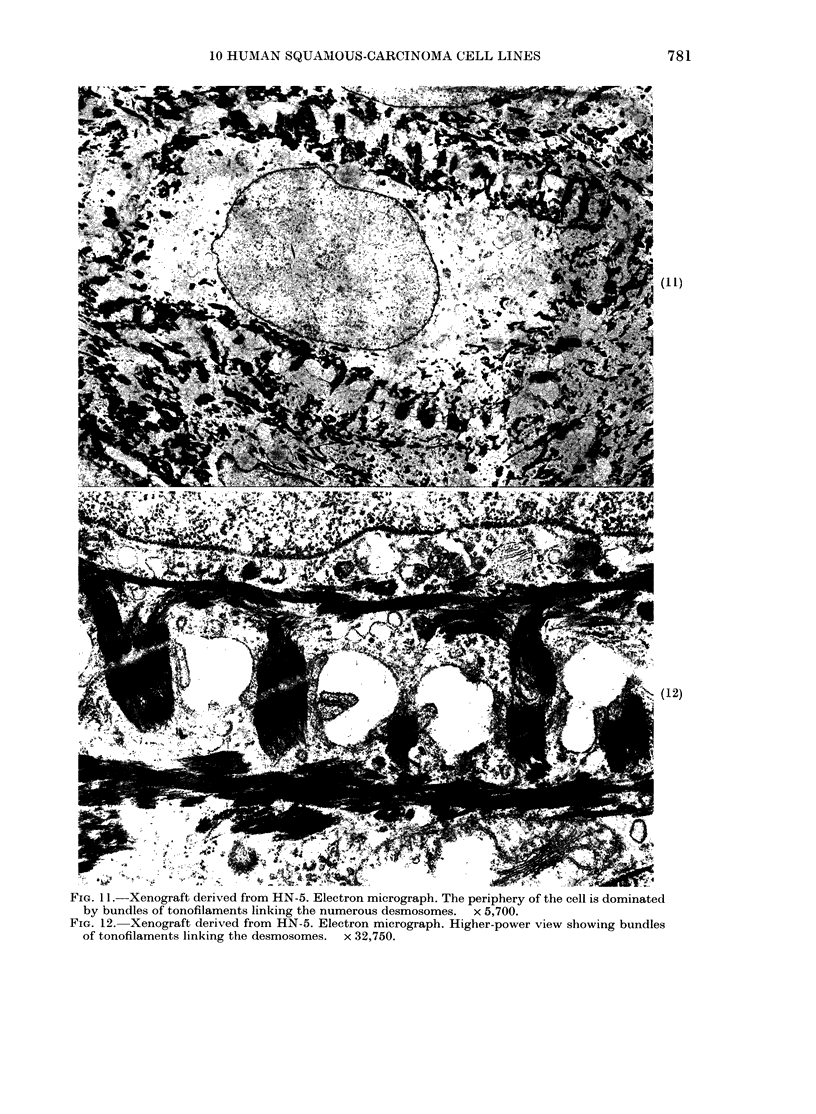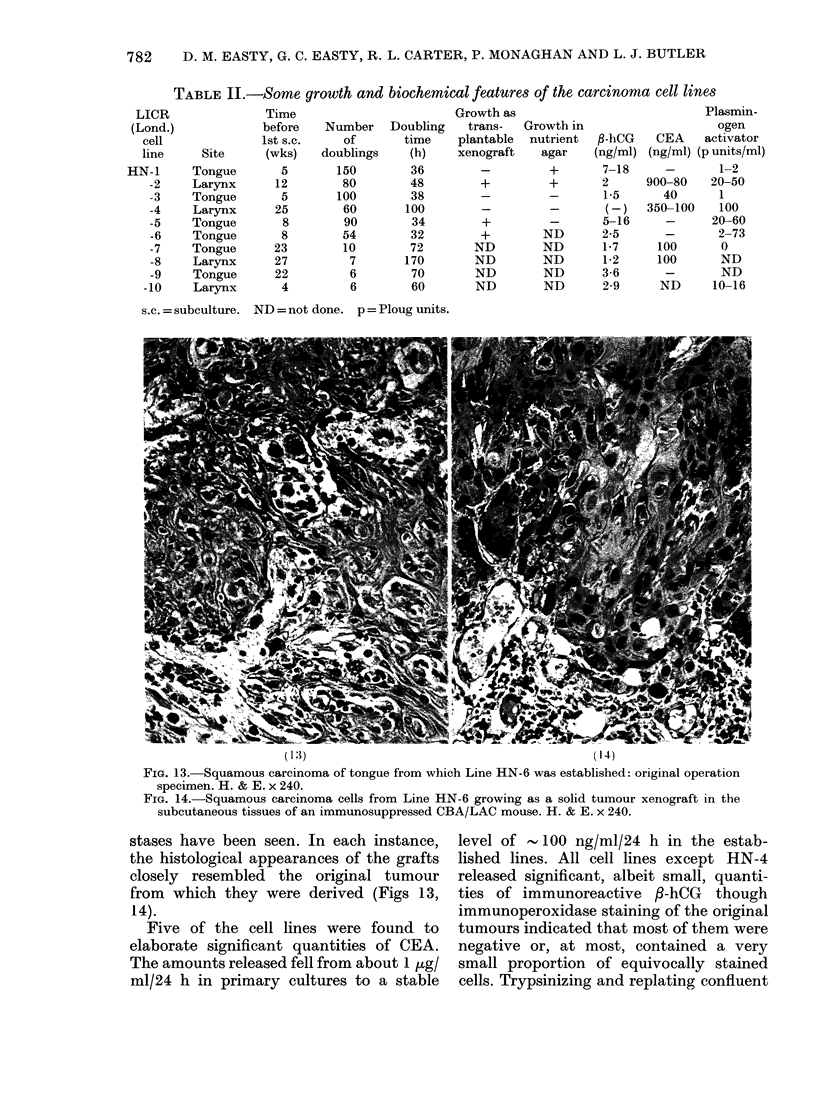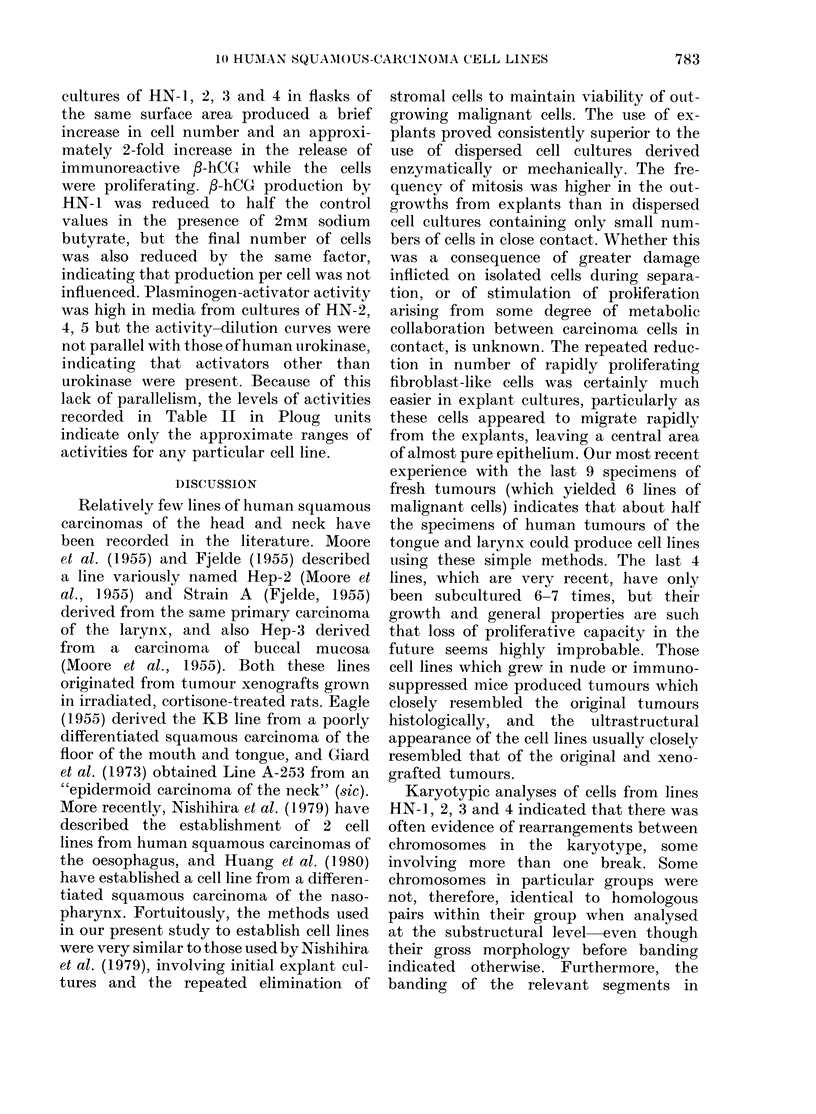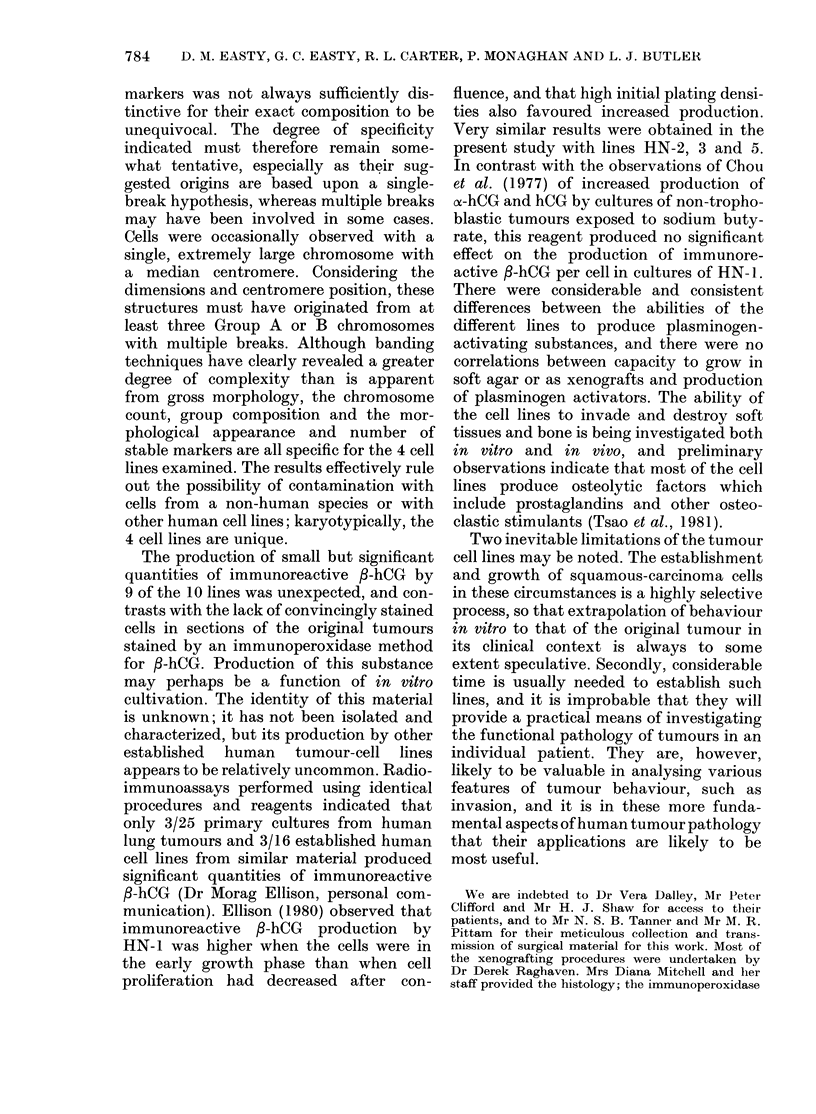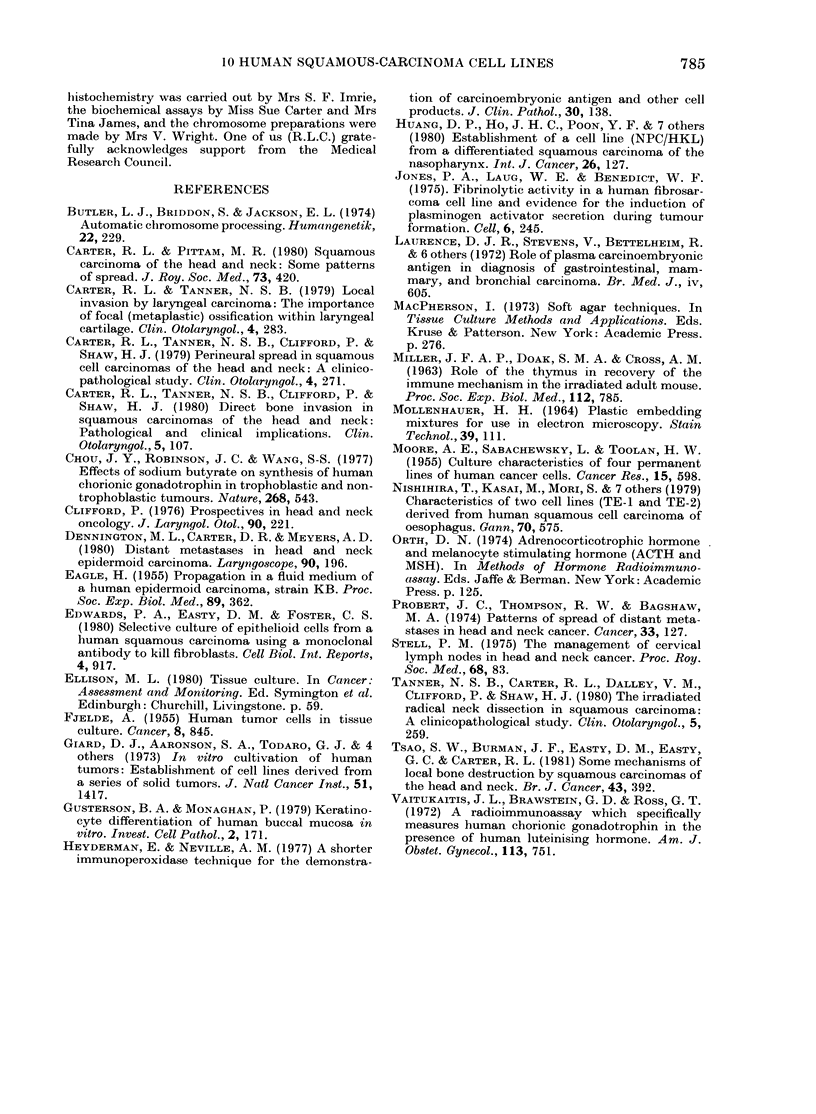# Ten human carcinoma cell lines derived from squamous carcinomas of the head and neck.

**DOI:** 10.1038/bjc.1981.115

**Published:** 1981-06

**Authors:** D. M. Easty, G. C. Easty, R. L. Carter, P. Monaghan, L. J. Butler

## Abstract

**Images:**


*Br. J. Cancer* (1981) **43,** 772

### TEN HUMAN CARCINOMA CELL LINES DERIVED FROM SQUAMOUS CARCINOMAS OF THE HEAD AND NECK

D. M. EASTY*, G. C. EASTY*, R. L. CARTER†, P. MONAGHAN* AND L. J. BUTLER‡

*From the *Ludwig Institute for Cancer Research (London Branch), Royal Marsden Hospital, Sutton, Surrey; †Institute of Cancer Research and Royal Marsden Hospital, Sutton, Surrey; and ‡Department of Cytogenetics, Queen Elizabeth Hospital for Children, Hackney, London*

Received 8 January 1981 Accepted 13 February 1981

**Summary.—Ten cell lines of human squamous carcinomas of the tongue and larynx have been established from surgical specimens removed from 36 unselected patients, in order to provide systems for investigating the invasive and tissue-destructive capacity of squamous carcinomas of the head and neck. The morphology, ultrastructure and growth characteristics of the 10 lines are described. Detailed cytogenetic analysis of the first 4 lines indicates that each is karyotypically unique, with no evidence of cross-contamination. Nine of the 10 cell lines secrete immunoreactive β human chorionic gonadotrophin (β-hCG) in the culture medium. No correlation was demonstrated between the ability of the cell lines to secrete plasminogen activator and their capacity to grow in soft agar or as xenografts in immune-deficient mice.**

SQUAMOUS CARCINOMAS of the head and neck are uncommon cancers in the United Kingdom, but they present formidable problems in clinical management. Most carcinomas of the lower lip, small cancers in the anterior part of the tongue, and carcinomas confined to the glottis carry a good prognosis; but survival in patients with tumours at other sites remains extremely poor (Stell, 1975; Clifford, 1976). Many tumours develop in or near vital anatomical structures in the upper respiratory or digestive systems, and their characteristic growth pattern is of diffuse infiltration of local soft tissues, invasion of lymphatics and blood vessels, destruction of contiguous bone and metastasis to regional lymph nodes. Evidence of more distant dissemination is often found at necropsy (Probert *et al.*, 1974; Dennington *et al.*, 1980) but clinically overt disease still tends to be concentrated in structures above the clavicles.

Detailed clinicopathological studies of the patterns of spread of squamous carcinomas of the head and neck into perineural spaces, bone and cartilage, and to regional lymph nodes, have been reported (Carter *et al.*, 1979, 1980; Carter & Tanner, 1979; Carter & Pittam, 1980; Tanner *et al.*, 1980); current investigations are concerned with some of the underlying mechanisms, with particular respect to tumour-associated destruction of soft tissues and bone (Tsao *et al.*, 1981). The information that can be obtained from examining fresh tumours is somewhat limited, and access to a number of continuous cell lines derived from squamous carcinomas of the head and neck would considerably facilitate investigations of the behaviour and properties of these tumours. Few such lines existed before this work began (Moore *et al.*, 1955; Eagle, 1955; Giard *et al.*, 1973; Nishihira *et al.*, 1979; Huang *et al.*, 1980). We describe here a relatively simple method which has produced 10 lines of squamous carcinomas of the head and neck, together with a brief account of their morphology, ultrastructure, karyo-

Address for reprints: Dr R. L. Carter, Haddow Laboratories, Royal Marsden Hospital, Sutton, Surrey SM2 5PX.

types, growth characteristics and biochemical activities.

### MATERIALS AND METHODS

*Tissue culture.*—Tumour tissue removed at the time of major surgery was immediately immersed in cold, sterile HEPES-buffered Medium 199 (Flow Laboratories) containing 150 i.u./ml benzyl penicillin (Glaxo), 100 $\mu$g/ml kanamycin (Bristol), 2·5 $\mu$g/ml Fungizone (Squibb) and 1 $\mu$g/ml of the anti-mycoplasma agent minocycline (Lederle). The tissue was usually received in the laboratory within 4 h of surgical removal, but occasionally it was kept overnight at 4°C without apparent loss of viability. Obviously necrotic or haemorrhagic regions and associated muscle and fat were removed under a dissecting microscope, and the remaining tissue reduced to 1–2 mm$^3$ fragments with a scalpel. About 8 fragments were transferred to a 25cm$^2$ plastic culture flask (Nunc, Gibco) containing not more than 2·5 ml Dulbecco Eagle's medium containing 10% foetal calf serum (FCS) and the same concentrations of antibiotics used for tissue collection. Each flask was gassed with 10% $CO_2$ in air to produce a pH of 7·4 and incubated at 37°C for a minimum of 4 days without disturbance, to facilitate attachment of the fragments to plastic. Some specimens were also incubated, with stirring, in solutions of 0·1% trypsin–versene or 0·1% collagenase to produce suspensions of dispersed cells for culture. Medium was renewed twice weekly once the tissue fragments or cells had become attached. All but two of the specimens produced outgrowths of proliferating fibroblasts in addition to epithelial cells. Fibroblast outgrowth was controlled by a combination of three methods: mechanical removal using a rubber-coated, fine pointed metal probe; exposure of cultures to complement-dependent, anti-human fibroblast monoclonal antibody (Edwards *et al.*, 1980); and selective detachment of fibroblasts by exposure to 0·1% trypsin–versene solution.

Subculture of primary cultures was never attempted until they were free of identifiable proliferating fibroblasts. Dilutions of cells at the first few subcultures were never more than 2-fold, as many trypsinized cells did not attach, and some of them may have been normal squamous epithelium of limited proliferative capacity. Aliquots of cells from early subcultures of each line were cryopreserved in HEPES-buffered Medium 199 containing 10% dimethylsulphoxide in liquid $N_2$. Growth of some of the cell lines in semi-solid agar was examined using the method of MacPherson (1973) and clones were derived from the first cell line by plating single cells in multi-well dishes containing 0·2 ml of Dulbecco Eagle's medium plus 10% FCS per well. Doubling times of established lines were determined by inoculating 25cm$^2$ culture flasks with $\sim 10^5$ cells and trypsinizing and counting the number of cells every 24 h for 5 days.

*Biochemical assays.*—Media which had been in contact with primary cultures and with $3\text{–}4 \times 10^6$ cells of established lines for 24 h were assayed for *carcinoembryonic antigen* (CEA) by the method of Laurence *et al.* (1972).

*$\beta$-subunit of human chorionic gonadotrophin* ($\beta$-hCG) was assayed using the SB6 antiserum provided by the National Institute of Arthritis. Metabolism and Diseases of the Digestive System (Vaitukaitis *et al.*, 1972) in a radioimmunoassay modified from the method of Orth (1974), in which polyethylene glycol was used to precipitate the bound hormone. The influence of cell proliferation on $\beta$-hCG secretion was examined in 4 cell lines by trypsinizing confluent cultures, replating all the cells in similar flasks, where they underwent a brief wave of proliferation, and assaying the medium, which was renewed every day for 7 days for $\beta$-hCG content. The influence of 2mM sodium butyrate on $\beta$-hCG secretion by one established cell line was examined by exposing replicate cultures to medium containing 2mM sodium butyrate, which was renewed every day for 5 days; the $\beta$-hCG content was then compared with control cultures re-fed every 24 h with medium containing no sodium butyrate. At the end of the experiment the cells were trypsinized and counted.

The release of *plasminogen activators* by the established cell lines was measured using the method of Jones *et al.* (1975) with $^{125}$I-labelled fibrin as substrate. Confluent cultures containing $3\text{–}4 \times 10^6$ cells were incubated for 24 h in Dulbecco Eagle's medium containing 10% FCS, in which the protease inhibitor, $\alpha_2$-macroglobulin had been inactivated by reduction of the pH of the serum to 3·0 for 2 h at room temperature. Plasminogen-activator activity of serial dilutions of the cell culture medium was measured and compared with the plasminogen-activator activity of dilutions of human urokinase (Leo Laboratories) varying from 25 to 0·1 Ploug units per ml in identical control medium.

*Xenografts.*—Nude or CBA/LAC female mice immunosuppressed by the method of Miller *et al.* (1963) were injected with $5 \times 10^6$ cells in 0·2 ml of Medium 199 s.c. and i.m. Cells of some of the lines were also grown in culture on small fragments of absorbable gelatin sponge (Sterispon, Allen and Hanbury Ltd) for several weeks, and the fragments implanted s.c. into the flanks of immunosuppressed mice. The animals were killed at various times and postmortem examinations were made. Part of any local nodule at the site of inoculation was transplanted into more immunosuppressed mice; the remainder, together with any other macroscopic lesions, were processed for light and sometimes electron microscopy.

*Histology and electron microscopy.*—Detailed histopathological examinations were made of all the original surgical specimens from which tissues were removed for culture, and all xenografted tumours. Standard techniques were used, supplemented with immunoperoxidase staining for $\beta$-hCG and CEA, using the method of Heyderman & Neville (1977).

Material for electron microscopy was fixed for 30 min at room temperature in 2% glutaraldehyde and post-fixed for 1 h in 1% osmium tetroxide. Both fixatives were phosphate-buffered (pH 7·2–7·4) and the osmotic pressure was adjusted to 350 m.osmoles by the addition of sucrose. Dehydration was carried out in a graded series of ethanol, and tissue samples embedded in Epon/Araldite (Mollenhauer, 1964) *via* propylene oxide. When the cultures grown in culture flasks were in absolute ethanol, the top of the flask was removed with a hot wire and the fixed layer of cells was cut into 5mm squares with a scalpel. The absolute ethanol was replaced by propylene oxide, and gentle agitation lifted the "squares" of cells from the surface of the flask. These were transferred to fresh propylene oxide and then embedded in Epon/ Araldite (Mollenhauer, 1964).

*Karyotypic analysis.*—Subconfluent cultures were monitored for evidence of mitotic activity, and when there were sufficient cells in various stages of mitosis, colchicine was added to the medium. The details of all subsequent procedures to produce air-dried preparations followed by trypsin G-banding were as previously described (Butler *et al.*, 1974) except that the automated system was not used; instead, final cell suspensions in 45% acetic acid were prepared, so that more than one slide was available from each culture.

### RESULTS

Of the 36 original specimens received, 7 were discarded within a few days or weeks because of rapid bacterial or fungal growth which was not eliminated by the antibiotics or antifungal agents included in the culture medium. Cell lines were established from 10 of the remaining 28 specimens, the last 6 from 9 consecutive resections—a success rate which probably reflects increasing expertise in handling this type of tissue in the laboratory. The relevant clinical and pathological details for the 10 patients from whom cell lines were established are summarized in Table I.

Only 2 of the lines grew *ab initio* without contamination with fibroblast-like cells (Fig. 1). The remainder produced rapidly proliferating fibroblast-like cells which, if not repeatedly reduced in number, either detached colonies of epithelial cells from the culture surface or grew over the epithelial cells and inhibited their proliferation. The number of fibroblasts was generally controlled by mechanical detachment using a rubber-coated probe, though both the antifibroblast monoclonal antibody (Edwards *et al.*, 1980) and selective detachment with trypsin–versene solution were used successfully to eliminate fibroblasts when large areas of epithelium were contaminated by a low proportion of fibroblasts. Explant cultures were found to be consistently superior to cultures obtained from individual cells isolated mechanically or by enzyme digestion. Epithelial-cell proliferation in primary cultures appeared to be highly dependent on cell–cell contact, which was maximal between epithelial cells migrating radially from explants. Mitoses were much rarer in cultures derived from cell suspensions,

TABLE I.—*Clinical and pathological findings in 10 patients from whom squamous-carcinoma cell lines have been established*

| LICR (Lond.) Cell line | Patient | Tumour | Initial TNM stage† | Treatment* |
|---|---|---|---|---|
| HN-1 | W.G. Male, 51 | Tongue Moderately differentiated | $T_2\ N_1\ M_0$ | Previous CT, RT. Hemiglossectomy, hemimandibulectomy, radical neck dissection. |
| -2 | M.K. Male, 49 | Larynx Poorly differentiated | $T_3\ N_0\ M_0$ | Previous RT. Total laryngectomy, radical neck dissection. |
| -3 | W.P. Male, 63 | Tongue Moderately differentiated | $T_3\ N_0\ M_0$ | Previous CT, RT. Hemiglossectomy, hemimandibulectomy, radical neck dissection. |
| -4 | M.E. Male, 57 | Larynx Moderately differentiated | $T_2\ N_0\ M_0$ | Previous CT, RT. Total laryngectomy |
| -5 | I.P. Male, 73 | Tongue Moderately differentiated | $T_2\ N_0\ M_0$ | Previous CT, RT. Hemiglossectomy, hemimandibulectomy, radical neck dissection. |
| -6 | L.B. Male, 54 | Tongue Well to moderately differentiated | $T_2\ N_0\ M_0$ | Previous CT, RT. Hemiglossectomy, suprahyoid block dissection. |
| -7 | P.B. Male, 57 | Tongue Poorly differentiated | $T_2\ N_0\ M_0$ | Previous CT, RT. Hemiglossectomy, radical neck dissection. |
| -8 | P.M. Male, 56 | Larynx Moderately differentiated | $T_2\ N_0\ M_0$ | Previous RT. Total laryngectomy. |
| -9 | P.M. Female, 67 | Tongue Moderately differentiated | $T_2\ N_0\ M_0$ | Previous RT. Hemiglossectomy, radical neck dissection. |
| -10 | P.M. Male, 57 | Larynx Poorly differentiated | $T_3\ N_0\ M_0$ | Previous RT. Total laryngectomy. |

* CT = chemotherapy; RT = radiotherapy.
† Assessed *clinically* at initial presentation.

where groups contained low numbers of cells in contact. In addition, the elimination of fibroblasts with minimal loss or damage to epithelial cells was much easier and more efficient when epithelial growth was confined to relatively few, circular foci. Many of the original tissue specimens included regions of non-malignant squamous epithelium, and initial contamination of primary explant cultures with normal epithelium was probably common. One specimen from a patient with squamous carcinoma of the larynx produced outgrowths of abundant ciliated epithelium which maintained synchronous ciliated activity for the first 5–6 weeks of culture. In general, the suspected normal epithelium consisted of cells which occupied a larger area and possessed a less granular cytoplasm than the malignant cells. They appeared to migrate from the explants in advance of malignant epithelium, formed somewhat different cellular arrangements at the leading edge (Figs 2, 3) and proliferated rapidly for the first few weeks before senescing and detaching by 7–9 weeks (Gusterson & Monaghan, 1979).

All the cell lines are epithelioid. Eight of them showed some density-dependent inhibition of mitosis, while the other 2 continued to divide vigorously even after confluence was obtained (Figs 4, 5). They

(1)

(2)

(3)

(4)

FIG. 1.—Primary explant of HN-1 after 5 days in culture, showing extensive cell migration and proliferation. Tripolar mitoses are present. No obvious contamination by fibroblasts. × 180.

FIG. 2.—Primary explant of HN-3 after 6 days in culture, showing presumptive normal epithelium with distinctive tangential leading edge. × 200.

FIG. 3.—Primary explant of HN-3 after 4 days in culture, showing typical squamous-carcinoma cells which continued to proliferate and did not senesce. × 180.

FIG. 4.—Subculture of HN-1 showing characteristic multilayering of cells, in contrast to most of the squamous-carcinoma lines. × 110.

FIG. 5.—A subculture of HN-5, forming mounds of cells after inoculating a flask with a hyperconfluent number of cells. Under normal subculturing conditions the cultures do not progress beyond a monolayer. ×180.

all shed material of cellular and subcellular size which, under phase-contrast microscopy and in sections of pelleted material, closely resembled keratinized epithelium lost from the surfaces of cultured squamous epithelium derived from the oral mucosa of normal individuals and of patients with non-malignant diseases (Gusterson & Monaghan, 1979). Tripolar mitoses, which are characteristic of many malignant tumours, were frequently observed in all the cell lines (see Fig. 1).

Electron-microscopic examination revealed that, when cultured on plastic surfaces, most of the cell lines showed some overlapping, did not form multilayers, and possessed very similar ultrastructural features: desmosomes were present but not abundant, cytoplasmic filaments were sparse, and there were occasional microvilli on cell surfaces exposed to the culture medium..

Three cell lines were significantly different from the others in their ultrastructural appearance. HN-1 readily formed multilayers from which desmosomes were completely absent, the only cell junctions consisting of occasional thickenings of adjacent cell membranes (Fig. 6). HN-7 formed rounded multicellular clumps resting on a cuboidal layer of cells. Fully formed desmosomes and cytoplasmic filaments were rare, and the cells were distinguished by their content of distended rough endoplasmic reticulum and irregular membranous structures (Figs 7, 8, 9). HN-5 was strikingly different from all the other lines, in that adjacent cells were linked by numerous desmosomes between which were small intercellular spaces. A dominant cytoplasmic feature was the large number of bundles of tonofilaments frequently interlinking the numerous desmosomes. Xenografts of HN-5 displayed the numerous desmosomes and tonofilaments which characterized most of the cells in the original tumour, and in the cultures from this tumour (Figs 10, 11, 12).

Karyotype analyses were carried out on cell lines HN-1 to 4. All of them were hyperdiploid, with mean numbers and ranges of counts as follows: HN-4 68 (52–92), HN-1 73 (50–92), HN-2 65 (59–72) and HN-3 63 (47–79). In most of the lines, most chromosome counts were clustered around the mean counts. Though the mean counts were not dissimilar the cells from each line differed both in the detailed composition of their chromosome groups and in the nature of their abnormal marker chromosomes. Most of the extra chromosomes in HN-4 occurred randomly in the C group (X—6–12) while in HN-1 the chromosome numbers were increased in the E & F groups. In HN-2 the A (12–14 per cell), C and E (8–12 per cell) groups, and in HN-3 the A (5–11 per cell), C (up to 33) and F (7–9) groups were increased. Notable reductions in other chromosome groups were also noted: G group in HN-4, D group in HN-1 and 2, and B group in HN-3.

FIG. 6.—Subculture of HN-1. Electron micrograph. The cells are rounded, with pleomorphic nuclei and prominent nucleoli. Desmosomes are not formed between adjacent cells, and the cytoplasm lacks bundles of tonofilaments. × 4,500.

All cell lines contained distinctive marker chromosomes which were often very large and readily distinguishable from each other. Their origin could not be correlated with changes in group composition, except in lines HN-1 and HN-2, in which reduced D groups were due to centric fusion, thus increasing the number in the A group. The cells from all lines except HN-3 contained one or more very small metacentric markers, the origin of which could not be identified even with banding. An analysis of G-banded material indicated that the large submetacentric chromosomes (2 per cell) found in HN-4, similar in shape to No. 2 but very much larger, consisted of an entire Chromosome No. 1 together with most of the long arm of No. 2. A second telocentric marker appeared to be a 4p–. The cells of HN-1 contained two large submetacentrics composed of an entire Chromosome 8 with a translocation of 2q to the long arms. A small "Dq+" marker probably consisted of Chromosome 22 with 4q, but in other cells a 13q+ marker was present. In HN-3 the single large acrocentric was apparently composed of Chromosome 10 and the distal part of the short arms of Chromosome No. 1. In HN-2, however, two somewhat similar markers resembling "Bp-" telocentrics were apparently 4p–, while two non-satellited acrocentrics were 18q+ chromosomes.

Some of the properties of the cell lines are summarized in Table II. The doubling times varied from 30 to 170 h. Lines HN-1 and HN-2 formed colonies in soft agar, while HN-3 ,4 and 5 did not. Manipulation of the culture conditions to facilitate the growth of HN-3, 4 and 5 in soft agar by the use of cell feeder layers, conditioned medium and added growth-promoting factors has not yet been attempted. Lines HN-2, 5, 6 but not HN-1, 3, 4 have been established as transplantable grafts in immunosuppressed or nude mice. The grafts grew locally as compact, partly encapsulated masses, adherent to the skin and the underlying body wall. No meta-

(7)

(8)

FIG. 7.—Subculture of HN-7. Electron micrograph. A region of cuboidal cells. Nuclei are irregular, and the cytoplasm contains vesicles of distended rough endoplasmic reticulum (arrow) and irregular electron-dense structures. × 6,050.

FIG. 8.—Subculture of HN-7. Electron micrograph. A rounded clump of cells. The cytoplasm contains large numbers of irregular electron-dense structures. × 2,550.

(9)

(10)

FIG. 9—. Subculture of HN-7. Electron micrograph. Higher-power view of cytoplasmic electron-dense structures showing membranous component. × 102,200.

FIG. 10.—Original surgical specimen of tumour from which Line HN-5 was derived. Electron micrograph. The cytoplasm of the cells is dominated by numerous bundles of tonofilaments. Desmosomes are also abundant. × 4,600.

(11)

(12)

FIG. 11.—Xenograft derived from HN-5. Electron micrograph. The periphery of the cell is dominated by bundles of tonofilaments linking the numerous desmosomes. × 5,700.

FIG. 12.—Xenograft derived from HN-5. Electron micrograph. Higher-power view showing bundles of tonofilaments linking the desmosomes. × 32,750.

TABLE II.—*Some growth and biochemical features of the carcinoma cell lines*

| LICR (Lond.) cell line | Site | Time before 1st s.c. (wks) | Number of doublings | Doubling time (h) | Growth as trans-plantable xenograft | Growth in nutrient agar | β-hCG (ng/ml) | CEA (ng/ml) | Plasmin-ogen activator (p units/ml) |
|---|---|---|---|---|---|---|---|---|---|
| HN-1 | Tongue | 5 | 150 | 36 | − | + | 7–18 | − | 1–2 |
| -2 | Larynx | 12 | 80 | 48 | + | + | 2 | 900–80 | 20–50 |
| -3 | Tongue | 5 | 100 | 38 | − | − | 1·5 | 40 | 1 |
| -4 | Larynx | 25 | 60 | 100 | − | − | (−) | 350–100 | 100 |
| -5 | Tongue | 8 | 90 | 34 | + | − | 5–16 | − | 20–60 |
| -6 | Tongue | 8 | 54 | 32 | + | ND | 2·5 | − | 2–73 |
| -7 | Tongue | 23 | 10 | 72 | ND | ND | 1·7 | 100 | 0 |
| -8 | Larynx | 27 | 7 | 170 | ND | ND | 1·2 | 100 | ND |
| -9 | Tongue | 22 | 6 | 70 | ND | ND | 3·6 | − | ND |
| -10 | Larynx | 4 | 6 | 60 | ND | ND | 2·9 | ND | 10–16 |

s.c. = subculture. ND = not done. p = Ploug units.

(13)

(14)

FIG. 13.—Squamous carcinoma of tongue from which Line HN-6 was established: original operation specimen. H. & E. × 240.

FIG. 14.—Squamous carcinoma cells from Line HN-6 growing as a solid tumour xenograft in the subcutaneous tissues of an immunosuppressed CBA/LAC mouse. H. & E. × 240.

stases have been seen. In each instance, the histological appearances of the grafts closely resembled the original tumour from which they were derived (Figs 13, 14).

Five of the cell lines were found to elaborate significant quantities of CEA. The amounts released fell from about 1 μg/ml/24 h in primary cultures to a stable level of ~100 ng/ml/24 h in the established lines. All cell lines except HN-4 released significant, albeit small, quantities of immunoreactive β-hCG though immunoperoxidase staining of the original tumours indicated that most of them were negative or, at most, contained a very small proportion of equivocally stained cells. Trypsinizing and replating confluent cultures of HN-1, 2, 3 and 4 in flasks of the same surface area produced a brief increase in cell number and an approximately 2-fold increase in the release of immunoreactive $\beta$-hCG while the cells were proliferating. $\beta$-hCG production by HN-1 was reduced to half the control values in the presence of 2mM sodium butyrate, but the final number of cells was also reduced by the same factor, indicating that production per cell was not influenced. Plasminogen-activator activity was high in media from cultures of HN-2, 4, 5 but the activity–dilution curves were not parallel with those of human urokinase, indicating that activators other than urokinase were present. Because of this lack of parallelism, the levels of activities recorded in Table II in Ploug units indicate only the approximate ranges of activities for any particular cell line.

### DISCUSSION

Relatively few lines of human squamous carcinomas of the head and neck have been recorded in the literature. Moore *et al.* (1955) and Fjelde (1955) described a line variously named Hep-2 (Moore *et al.*, 1955) and Strain A (Fjelde, 1955) derived from the same primary carcinoma of the larynx, and also Hep-3 derived from a carcinoma of buccal mucosa (Moore *et al.*, 1955). Both these lines originated from tumour xenografts grown in irradiated, cortisone-treated rats. Eagle (1955) derived the KB line from a poorly differentiated squamous carcinoma of the floor of the mouth and tongue, and Giard *et al.* (1973) obtained Line A-253 from an "epidermoid carcinoma of the neck" (*sic*). More recently, Nishihira *et al.* (1979) have described the establishment of 2 cell lines from human squamous carcinomas of the oesophagus, and Huang *et al.* (1980) have established a cell line from a differentiated squamous carcinoma of the nasopharynx. Fortuitously, the methods used in our present study to establish cell lines were very similar to those used by Nishihira *et al.* (1979), involving initial explant cultures and the repeated elimination of stromal cells to maintain viability of outgrowing malignant cells. The use of explants proved consistently superior to the use of dispersed cell cultures derived enzymatically or mechanically. The frequency of mitosis was higher in the outgrowths from explants than in dispersed cell cultures containing only small numbers of cells in close contact. Whether this was a consequence of greater damage inflicted on isolated cells during separation, or of stimulation of proliferation arising from some degree of metabolic collaboration between carcinoma cells in contact, is unknown. The repeated reduction in number of rapidly proliferating fibroblast-like cells was certainly much easier in explant cultures, particularly as these cells appeared to migrate rapidly from the explants, leaving a central area of almost pure epithelium. Our most recent experience with the last 9 specimens of fresh tumours (which yielded 6 lines of malignant cells) indicates that about half the specimens of human tumours of the tongue and larynx could produce cell lines using these simple methods. The last 4 lines, which are very recent, have only been subcultured 6–7 times, but their growth and general properties are such that loss of proliferative capacity in the future seems highly improbable. Those cell lines which grew in nude or immunosuppressed mice produced tumours which closely resembled the original tumours histologically, and the ultrastructural appearance of the cell lines usually closely resembled that of the original and xenografted tumours.

Karyotypic analyses of cells from lines HN-1, 2, 3 and 4 indicated that there was often evidence of rearrangements between chromosomes in the karyotype, some involving more than one break. Some chromosomes in particular groups were not, therefore, identical to homologous pairs within their group when analysed at the substructural level—even though their gross morphology before banding indicated otherwise. Furthermore, the banding of the relevant segments in markers was not always sufficiently distinctive for their exact composition to be unequivocal. The degree of specificity indicated must therefore remain somewhat tentative, especially as their suggested origins are based upon a single-break hypothesis, whereas multiple breaks may have been involved in some cases. Cells were occasionally observed with a single, extremely large chromosome with a median centromere. Considering the dimensions and centromere position, these structures must have originated from at least three Group A or B chromosomes with multiple breaks. Although banding techniques have clearly revealed a greater degree of complexity than is apparent from gross morphology, the chromosome count, group composition and the morphological appearance and number of stable markers are all specific for the 4 cell lines examined. The results effectively rule out the possibility of contamination with cells from a non-human species or with other human cell lines; karyotypically, the 4 cell lines are unique.

The production of small but significant quantities of immunoreactive $\beta$-hCG by 9 of the 10 lines was unexpected, and contrasts with the lack of convincingly stained cells in sections of the original tumours stained by an immunoperoxidase method for $\beta$-hCG. Production of this substance may perhaps be a function of *in vitro* cultivation. The identity of this material is unknown; it has not been isolated and characterized, but its production by other established human tumour-cell lines appears to be relatively uncommon. Radioimmunoassays performed using identical procedures and reagents indicated that only 3/25 primary cultures from human lung tumours and 3/16 established human cell lines from similar material produced significant quantities of immunoreactive $\beta$-hCG (Dr Morag Ellison, personal communication). Ellison (1980) observed that immunoreactive $\beta$-hCG production by HN-1 was higher when the cells were in the early growth phase than when cell proliferation had decreased after confluence, and that high initial plating densities also favoured increased production. Very similar results were obtained in the present study with lines HN-2, 3 and 5. In contrast with the observations of Chou *et al.* (1977) of increased production of $\alpha$-hCG and hCG by cultures of non-trophoblastic tumours exposed to sodium butyrate, this reagent produced no significant effect on the production of immunoreactive $\beta$-hCG per cell in cultures of HN-1. There were considerable and consistent differences between the abilities of the different lines to produce plasminogen-activating substances, and there were no correlations between capacity to grow in soft agar or as xenografts and production of plasminogen activators. The ability of the cell lines to invade and destroy soft tissues and bone is being investigated both *in vitro* and *in vivo*, and preliminary observations indicate that most of the cell lines produce osteolytic factors which include prostaglandins and other osteoclastic stimulants (Tsao *et al.*, 1981).

Two inevitable limitations of the tumour cell lines may be noted. The establishment and growth of squamous-carcinoma cells in these circumstances is a highly selective process, so that extrapolation of behaviour *in vitro* to that of the original tumour in its clinical context is always to some extent speculative. Secondly, considerable time is usually needed to establish such lines, and it is improbable that they will provide a practical means of investigating the functional pathology of tumours in an individual patient. They are, however, likely to be valuable in analysing various features of tumour behaviour, such as invasion, and it is in these more fundamental aspects of human tumour pathology that their applications are likely to be most useful.

We are indebted to Dr Vera Dalley, Mr Peter Clifford and Mr H. J. Shaw for access to their patients, and to Mr N. S. B. Tanner and Mr M. R. Pittam for their meticulous collection and transmission of surgical material for this work. Most of the xenografting procedures were undertaken by Dr Derek Raghaven. Mrs Diana Mitchell and her staff provided the histology; the immunoperoxidase histochemistry was carried out by Mrs S. F. Imrie, the biochemical assays by Miss Sue Carter and Mrs Tina James, and the chromosome preparations were made by Mrs V. Wright. One of us (R.L.C.) gratefully acknowledges support from the Medical Research Council.

### REFERENCES

Butler, L. J., Briddon, S. & Jackson, E. L. (1974) Automatic chromosome processing. *Humangenetik*, **22**, 229.

Carter, R. L. & Pittam, M. R. (1980) Squamous carcinoma of the head and neck: Some patterns of spread. *J. Roy. Soc. Med.*, **73**, 420.

Carter, R. L. & Tanner, N. S. B. (1979) Local invasion by laryngeal carcinoma: The importance of focal (metaplastic) ossification within laryngeal cartilage. *Clin. Otolaryngol.*, **4**, 283.

Carter, R. L., Tanner, N. S. B., Clifford, P. & Shaw, H. J. (1979) Perineural spread in squamous cell carcinomas of the head and neck: A clinicopathological study. *Clin. Otolaryngol.*, **4**, 271.

Carter, R. L., Tanner, N. S. B., Clifford, P. & Shaw, H. J. (1980) Direct bone invasion in squamous carcinomas of the head and neck: Pathological and clinical implications. *Clin. Otolaryngol.*, **5**, 107.

Chou, J. Y., Robinson, J. C. & Wang, S-S. (1977) Effects of sodium butyrate on synthesis of human chorionic gonadotrophin in trophoblastic and non-trophoblastic tumours. *Nature*, **268**, 543.

Clifford, P. (1976) Prospectives in head and neck oncology. *J. Laryngol. Otol.*, **90**, 221.

Dennington, M. L., Carter, D. R. & Meyers, A. D. (1980) Distant metastases in head and neck epidermoid carcinoma. *Laryngoscope*, **90**, 196.

Eagle, H. (1955) Propagation in a fluid medium of a human epidermoid carcinoma, strain KB. *Proc. Soc. Exp. Biol. Med.*, **89**, 362.

Edwards, P. A., Easty, D. M. & Foster, C. S. (1980) Selective culture of epithelioid cells from a human squamous carcinoma using a monoclonal antibody to kill fibroblasts. *Cell Biol. Int. Reports*, **4**, 917.

Ellison, M. L. (1980) Tissue culture. In *Cancer: Assessment and Monitoring*. Ed. Symington *et al.* Edinburgh: Churchill, Livingstone. p. 59.

Fjelde, A. (1955) Human tumor cells in tissue culture. *Cancer*, **8**, 845.

Giard, D. J., Aaronson, S. A., Todaro, G. J. & 4 others (1973) *In vitro* cultivation of human tumors: Establishment of cell lines derived from a series of solid tumors. *J. Natl Cancer Inst.*, **51**, 1417.

Gusterson, B. A. & Monaghan, P. (1979) Keratinocyte differentiation of human buccal mucosa *in vitro*. *Invest. Cell Pathol.*, **2**, 171.

Heyderman, E. & Neville, A. M. (1977) A shorter immunoperoxidase technique for the demonstration of carcinoembryonic antigen and other cell products. *J. Clin. Pathol.*, **30**, 138.

Huang, D. P., Ho, J. H. C., Poon, Y. F. & 7 others (1980) Establishment of a cell line (NPC/HKL) from a differentiated squamous carcinoma of the nasopharynx. *Int. J. Cancer*, **26**, 127.

Jones, P. A., Laug, W. E. & Benedict, W. F. (1975). Fibrinolytic activity in a human fibrosarcoma cell line and evidence for the induction of plasminogen activator secretion during tumour formation. *Cell*, **6**, 245.

Laurence, D. J. R., Stevens, V., Bettelheim, R. & 6 others (1972) Role of plasma carcinoembryonic antigen in diagnosis of gastrointestinal, mammary, and bronchial carcinoma. *Br. Med. J.*, iv, 605.

MacPherson, I. (1973) Soft agar techniques. In *Tissue Culture Methods and Applications*. Eds. Kruse & Patterson. New York: Academic Press. p. 276.

Miller, J. F. A. P., Doak, S. M. A. & Cross, A. M. (1963) Role of the thymus in recovery of the immune mechanism in the irradiated adult mouse. *Proc. Soc. Exp. Biol. Med.*, **112**, 785.

Mollenhauer, H. H. (1964) Plastic embedding mixtures for use in electron microscopy. *Stain Technol.*, **39**, 111.

Moore, A. E., Sabachewsky, L. & Toolan, H. W. (1955) Culture characteristics of four permanent lines of human cancer cells. *Cancer Res.*, **15**, 598.

Nishihira, T., Kasai, M., Mori, S. & 7 others (1979) Characteristics of two cell lines (TE-1 and TE-2) derived from human squamous cell carcinoma of oesophagus. *Gann*, **70**, 575.

Orth, D. N. (1974) Adrenocorticotrophic hormone and melanocyte stimulating hormone (ACTH and MSH). In *Methods of Hormone Radioimmunoassay*. Eds. Jaffe & Berman. New York: Academic Press. p. 125.

Probert, J. C., Thompson, R. W. & Bagshaw, M. A. (1974) Patterns of spread of distant metastases in head and neck cancer. *Cancer*, **33**, 127.

Stell, P. M. (1975) The management of cervical lymph nodes in head and neck cancer. *Proc. Roy. Soc. Med.*, **68**, 83.

Tanner, N. S. B., Carter, R. L., Dalley, V. M., Clifford, P. & Shaw, H. J. (1980) The irradiated radical neck dissection in squamous carcinoma: A clinicopathological study. *Clin. Otolaryngol.*, **5**, 259.

Tsao, S. W., Burman, J. F., Easty, D. M., Easty, G. C. & Carter, R. L. (1981) Some mechanisms of local bone destruction by squamous carcinomas of the head and neck. *Br. J. Cancer*, **43**, 392.

Vaitukaitis, J. L., Brawstein, G. D. & Ross, G. T. (1972) A radioimmunoassay which specifically measures human chorionic gonadotrophin in the presence of human luteinising hormone. *Am. J. Obstet. Gynecol.*, **113**, 751.